# Complete Nucleotide Sequence of a *Citrobacter freundii* Plasmid Carrying KPC-2 in a Unique Genetic Environment

**DOI:** 10.1128/genomeA.01157-14

**Published:** 2014-11-13

**Authors:** Yancheng Yao, Can Imirzalioglu, Torsten Hain, Martin Kaase, Soeren Gatermann, Martin Exner, Martin Mielke, Anja Hauri, Yolanta Dragneva, Rita Bill, Constanze Wendt, Angela Wirtz, Eugen Domann, Trinad Chakraborty

**Affiliations:** a Institute for Medical Microbiology, German Centre of Infection Research, Justus-Liebig-University Giessen, Giessen, Germany; b Institute for Hygiene and Medical Microbiology, Ruhr University, Bochum, Germany; c Institute for Hygiene and Public Health, University of Bonn, Bonn, Germany; d Robert Koch Institute, Berlin, Germany; e Hessisches Landesprüfungs- und Untersuchungsamt im Gesundheitswesen, Dillenburg, Germany.; f Gesundheits- und Pflegezentrum Rüsselsheim, Rüsselsheim, Germany; g Labor Limbach, Heidelberg, Germany; h Hessisches Ministerium für Soziales und Integration, Wiesbaden, Germany

## Abstract

The complete and annotated nucleotide sequence of a 54,036-bp plasmid harboring a *bla*_KPC-2_ gene that is clonally present in *Citrobacter* isolates from different species is presented. The plasmid belongs to incompatibility group N (IncN) and harbors the class A carbapenemase KPC-2 in a unique genetic environment.

## GENOME ANNOUNCEMENT

Carbapenems remain the most effective antibiotics for the treatment of serious infections caused by multi-resistant Gram-negative bacteria producing extended-spectrum β-lactamases (ESBL). The rise of carbapenem-resistant Gram-negative bacteria is increasingly being reported and is now a matter of great clinical concern. Carbapenem resistance in *Enterobacteriaceae* is mainly due to the production of carbapenemases, the most common of which is the *K. pneumoniae* carbapenemase (KPC) family of enzymes (1–3).

Recently, isolation of a cluster of carbapenem-resistant *Citrobacter* species was reported from a single hospital environment in southern Hesse, Germany (4). Most isolates were typed as *C. freundii* but several isolates of carbapenem-resistant *C. amalonaticus*, *C. braakii*, and *C. koseri* were also detected. *Citrobacter* species are environmental pathogens that can colonize the intestinal tract of humans and animals. They are generally considered low-grade pathogens that rarely cause infections. However, these bacteria have been associated with a wide spectrum of infections involving the central nervous system and the gastrointestinal, urinary, and respiratory tracts (5).

Preliminary characterization revealed that all isolates harbored a nonconjugable *bla*_KPC-2_ gene. In order to determine the genetic localization of the KPC-2 we determined the genome sequence of 11 representative strains (8 *C. freundii*, 1 *C. amalonaticus*, 1 *C. braakii*, 1 *C. koseri*). DNA sequencing libraries were prepared using the Nextera XT kit (Illumina, San Diego) according to the manufacturer’s instructions. Individually tagged libraries were sequenced as a part of a flowcell as 2×300 base paired-end reads using the Illumina MiSeq platform (Illumina, San Diego). A total of 12,447,167,642 sequences were produced and the sequences from each isolate were separately assembled using CLC Genomics Workbench version 7.0.4. We identified contigs harboring *bla*_KPC-2_ by using ResFinder (http://cge.cbs.dtu.dk/services/ResFinder/) and assembled the flanking sequences to generate a closed contig comprised of 54,036 bp with 82 coding sequences (CDS) (6). Open reading frame (ORF) finding and gene annotation was done by using RAST (http://rast.nmpdr.org/) and a genetic map of the resulting contigs was generated with MAUVE (7, 8) and with the plasmid reference nucleotide sequence of pKPC_FCF/3SP (accession no. CP004367.2). Further analysis revealed that *bla*_KPC-2_ is located on an IncN plasmid and inserted in a region between the *tra*I and *tra*G genes (9–11). The *bla*_KPC-2_ gene is part of a 9,571-bp insertion with a unique genetic environment comprising at one end of a Tn4401 element with the ISKpn6 and Kpc-2 genes and an adjacent Tn3-like segment (12–14), harboring a *bla*_TEM1b_, ISCfr1, and *aac3-IId* genes flanked by a 137 bp direct repeat. All sequenced strains harbor genetically identical plasmids, suggesting its horizontal spread among the different *Citrobacter* species. The plasmid, derived from *C. freundii* isolate Cfr08698 encoding *bla*_KPC-2_, was designated pCfr-08698KPC-2.

### Nucleotide sequence accession number.

The nucleotide sequence of the *C. freundii* plasmid carrying *bla*_KPC-2_ has been deposited in the EMBL database under accession no. LN610760.
